# Clinical Characteristics and Outcomes for Solitary Fibrous Tumor (SFT): A Single Center Experience

**DOI:** 10.1371/journal.pone.0140362

**Published:** 2015-10-15

**Authors:** Nicholas DeVito, Evita Henderson, Gang Han, Damon Reed, Marilyn M. Bui, Robert Lavey, Lary Robinson, Jonathan S. Zager, Ricardo J. Gonzalez, Vernon K. Sondak, G. Douglas Letson, Anthony Conley

**Affiliations:** 1 Sarcoma Department, H. Lee Moffitt Cancer Center and Research Institute, Tampa, Florida, United States of America; 2 Anatomic Pathology Department, H. Lee Moffitt Cancer Center and Research Institute, Tampa, Florida, United States of America; 3 Biostatistics Department, Yale University School of Public Health, New Haven, Connecticut, United States of America; 4 Radiation Oncology Department, H. Lee Moffitt Cancer Center and Research Institute, Tampa, Florida, United States of America; 5 Thoracic Oncology Department, H. Lee Moffitt Cancer Center and Research Institute, Tampa, Florida, United States of America; 6 Cutaneous Oncology Department, H. Lee Moffitt Cancer Center and Research Institute, Tampa, Florida, United States of America; Johns Hopkins University, UNITED STATES

## Abstract

Solitary fibrous tumor (SFT) is a mesenchymal neoplasm of fibrous origin. The 2013 WHO classification of soft tissue tumors defines malignant forms as hypercellular, mitotically active (>4 mitosis/10 high-power fields), with cytological atypia, tumor necrosis, and/or infiltrative margins. With an IRB-approved protocol, we investigated patient records and clinicopathologic data from our Sarcoma Database to describe the clinical characteristics of both benign and malignant SFT. All pathology specimens were reviewed by two pathologists. Descriptive statistics and univariate/multivariate survival analysis were performed. Patient records and Social Security Death Index were used to evaluate vital status. Of 82 patients, 47 (57%) were women and 73 (89%) were Caucasian. Median age was 62 years (range, 20 to 89). Thirty-two (39%) patients succumbed to the disease. Primary tumor site was lung/pleura in 28 (34%), abdomen/pelvis in 23 (28%), extremity in 13 (16%), and head/neck in 9 (11%) patients. Pathology was described as benign in 42 (51%) and malignant in 40 (49%) patients. Compared to benign SFT, malignant histology is associated with larger tumor size, higher mitotic counts, metastatic disease at diagnosis, and greater use of chemotherapy and radiation therapy. Gender, age, and tumor site were not significantly different between benign and malignant subtypes. By univariate analysis, only benign vs. malignant variant and complete resection positively impacted overall survival (P = 0.02 and P<0.0001, respectively). In the multivariable analysis of overall survival, receiving chemotherapy or not receiving surgery were two variables significantly associated with higher failure rate in overall survival: patients with chemotherapy vs. no chemotherapy (P = 0.003, HR = 4.55, with 95% CI: 1.68–12.34) and patients without surgery vs. with surgery (P = 0.005, HR = 25.49, with 95% CI: 2.62–247.57). Clear survival differences exist between benign and malignant SFT. While surgery appears to be the best treatment option for benign and malignant SFT, better systemic therapies are needed to improve outcomes of patients with metastatic, malignant SFT.

## Introduction

Solitary fibrous tumor (SFT) is a mesenchymal tumor of fibroblastic type that is poorly understood. Originally regarded as separate entities, SFT and hemangiopericytoma are now considered one neoplasm. In the 2013 WHO classification of soft tissue tumors, the terminology is unified under SFT only. [1] These tumors exhibit fibroblastic-type differentiation and typically affect adults from ages 20 to 70 years. [1, 2]SFT commonly affects the pleura, peritoneum, meninges, and lower extremities, but they can be found in any body site. [3–5] Approximately 12–22% are found to be malignant [6]; however, the histological definitions that suggest a tendency toward malignancy are not well defined. England et al. determined benign versus malignant characteristics in pleural SFT, followed by Vallat-Decouvelaere et al. who first distinguished malignant from benign variants of extrapleural SFT on the basis of nuclear atypia, hypercellularity, greater than 4 mitosis/10 high-power fields, and necrosis. [7, 8] The 2013 WHO classification of soft tissue tumors further defines malignant forms as hypercellular, mitotically active (>4 mitosis/10 high-power fields), with cytological atypia, tumor necrosis, and/or infiltrative margins. [1]

Surgery is the treatment of choice for local disease, with 10-year survival rates reported to be between 54% and 89% after complete surgical resection with clear margins. [6, 9, 10] In patients with distant metastases or local recurrences after resection, options are limited and have mostly been studied in meningeal disease. Radiotherapy is often used to improve local control, and chemotherapy is often a final effort. Many patients do not respond to chemotherapy. [11–15] One recent retrospective study evaluated the efficacy of temozolomide and bevacizumab to treat advanced/metastatic SFT and noted that response, as defined by Choi criteria, correlates with improved progression-free survival compared to non-responders. The authors of that study concluded that a prospective trial is warranted in these patients. [16] Additionally, a prospective trial was presented at AACR in 2010 that examined the molecular characteristics and targeted therapeutics for patients with SFT resistant to conventional chemotherapies. [17] This study examined the expression of platelet-derived growth factor receptor and insulin-like growth factor I receptor/insulin receptor in 11 patients with SFT and their treatment response to sunitinib maleate and figitumumab, which target each of these receptors, respectively. Further study is still needed, but these therapies showed promise in these patients. [18]

We hypothesize that identifying the clinical differences between benign and malignant SFT will allow clinicians to define better therapy strategies for each subset. There has been recent progress in understanding the molecular basis of this disease, which defined the translocation of *NAB2* and *STAT6*. NAB2, a transcriptional repressor of EGR1 genes, is turned into a transcriptional activator when it undergoes fusion with the activating domain of the cell signaling molecule STAT6. [17–19] There have been some clinicopathological correlation studies of this entity. [3, 4, 6, 9, 11–14, 20] However, our present study is the largest one thus far to investigate these differences. This retrospective study was conducted to further assess histologic characteristics, therapy, and other factors versus outcome in patients with benign and malignant SFT.

## Materials and Methods

### Ethics Statement

This retrospective review was conducted with a protocol approved by the Institutional Review Board and the Scientific Review Board at Moffitt Cancer Center. Data was analyzed anonymously.

### Patients

Patients with histologically confirmed SFT treated at the Moffitt Cancer Center during a 17-year-period (from 1993 to 2010) were first identified through the Sarcoma Database and confirmed through the institutional electronic medical record. This retrospective review was conducted with a protocol approved by the Institutional Review Board and the Scientific Review Board. The clinical data collected and analyzed anonymously included age of the patient, gender, ethnicity, vital status, date of last follow up, age of presentation, primary tumor site, date and site of metastases, and chemotherapeutic, surgical, and radiotherapeutic interventions (available in Supporting Information, **S1 Dataset**).

### Pathological Review

**Fig 1:**
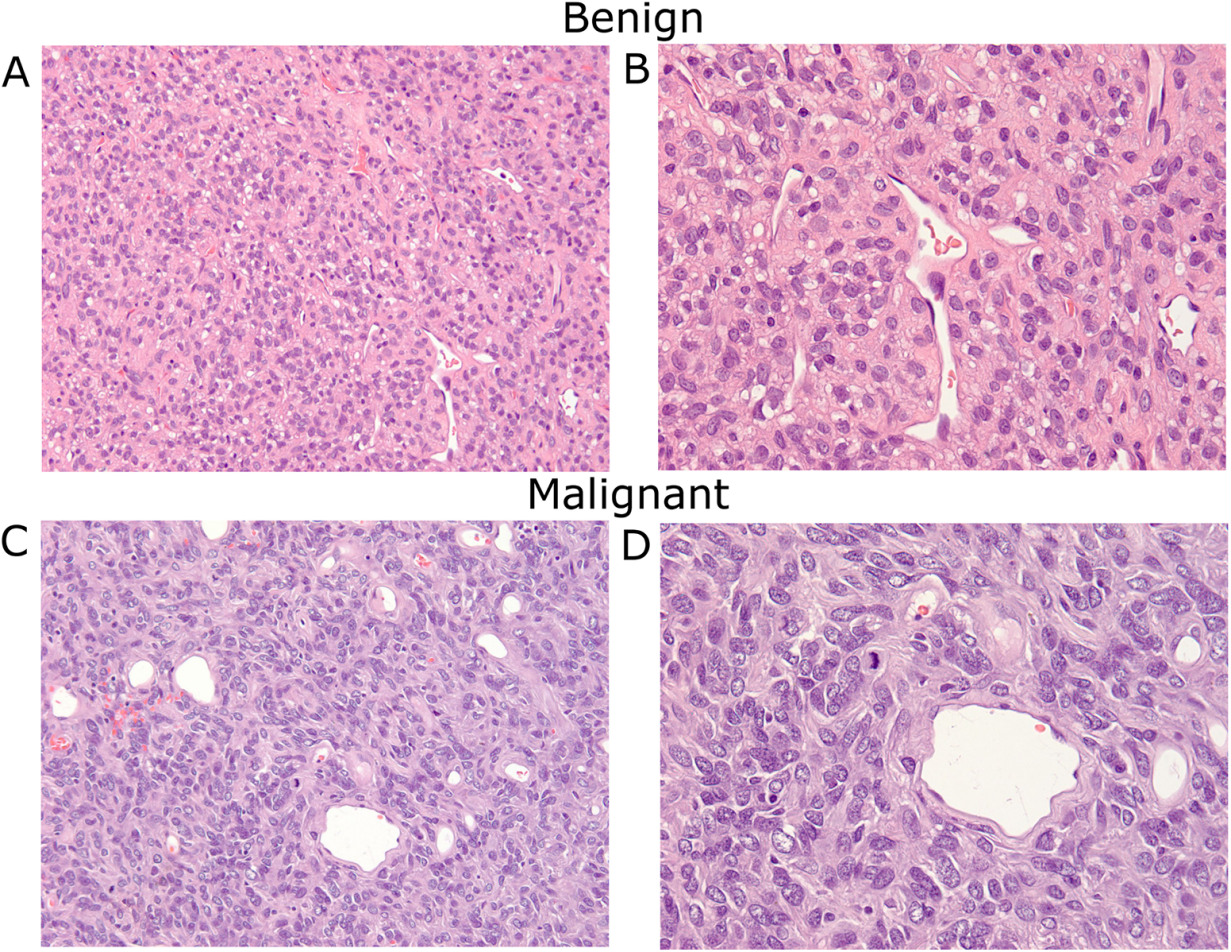
Histologic illustration of benign and malignant SFT. A: Benign SFT. The tumor shows a patternless architecture composed of spindle cells with hyalinized stroma and thin-walled branching vessels (H&E stain; x200 magnification). B: Benign SFT. The spindle tumor cells have vesicular nuclei without significant cytological atypia, mitosis, and necrosis (H&E stain; x400 magnification). C: Malignant SFT. The tumor has similar architecture; however, cells exhibit marked cytological atypia, including nuclear pleomorphism and increased mitotic activity (H&E stain; x200 magnification). D: Malignant SFT showing atypical mitotic tumor (H&E stain; x400 magnification).

The pathological diagnosis of all patients was established at presentation to Moffitt Cancer Center. Experienced sarcoma pathologists (E.H. and M.B.) re-reviewed and confirmed each case for this study. Diagnostic criteria for malignant SFT included use of the WHO publication of 2002, which has remained unchanged for the 2013 updated version, as well as the criteria established by English et al and Vallat-Decouvelaere. [1, 7, 8] Attention was placed on the mitotic count of each specimen separate from other variables during the statistical analysis, as this is an important indicator for histological diagnosis of malignancy and the aggressiveness of the tumor. Histological status included verification of tumor diagnosis and evaluation of whether it was benign or malignant. Examples of benign and malignant SFT are illustrated in Fig 1.

### Statistical Analysis

Descriptive statistics were utilized to summarize the clinical data. Recurrence-free survival was defined from the date of surgical treatment to the development of recurrent disease. Overall survival was defined from the date of diagnosis to the date of death by any cause. Univariate analyses for recurrence-free survival and overall survival were performed with the Kaplan-Meier method, and survival differences were evaluated using the log-rank test. Multivariate survival analysis was performed with Cox proportional hazard regression models. Backward elimination was utilized to determine the significance of each variable in this analysis. Survival probabilities at years 1, 3, and 5 and associated hazard ratios were reported in the usual manner. The statistical analysis was carried out with SAS software, version 9.3 (SAS Corporation, Cary, NC).

## Results

### Clinical Characteristics

Of 82 patients, 47 (57%) were women and 73 (89%) were Caucasian. Median age was 62 years (range, 20–89 years). The median follow-up was 55.3 months. Primary tumor site was lung/pleura in 28 (34%), abdomen/pelvis in 23 (28%), extremity in 13 (16%), and head/neck in 9 (11%) patients. Pathology was described and reconfirmed as benign in 42 (51%) and as malignant in 40 (49%) patients. A second review noted 3 patients with benign tumors that were morphologically atypical, which were short of diagnosis of malignancy and thus remained classified as benign but did exhibited cytological atypia and hypercellularity. Among these, 2 patients with benign/atypical tumors had recurrence and 1 died. Eleven (26%) of the 42 patients with benign disease had tumor recurrence, 6 of which were local and 5 distant. Of note, 27% of all SFT patients had other malignancies. There were no statistical differences between either group (see **Table 1** for details). Four of the patients with benign disease had concurrent advanced secondary malignancies, two with pancreatic cancer, one with prostate cancer, and one with renal cell carcinoma.

**Table 1:** Frequency (percentage) or median (*interquartile range) of patient characteristics for benign, malignant, and the whole groups.

	Benign n (%)	Malignant n (%)	Total n (%)	P Value
Number	42 (51%)	40 (49%)	82 (100%)	
**Age at diagnosis, years (Median)**	57.5 (47,77)*	63 (53.5, 72.5)*	61.5 (49,73)*	0.767
**Gender**				0.354
Male	20 (48%)	15 (38%)	35 (43%)	
Female	22 (52%)	25 (62%)	47 (57%)	
**Tumor size**				0.017
<5 cm	14 (38%)	4 (11%)	18 (25%)	
5–10 cm	8 (22%)	16 (46%)	25 (34%)	
>10 cm	15 (40%)	15 (43%)	30 (41%)	
**Mitotic count**				<0.001
<4/10 HPF	39 (98%)	9 (23%)	48 (60%)	
4-10/10 HPF	1 (2%)	25 (62%)	26 (33%)	
>10/10 HPF	0	6 (15)	6 (7%)	
**CD34 status**				0.230
Yes	30 (88%)	30 (81%)	60 (85%)	
No	4 (12%)	7 (19%)	11 (15%)	
**Primary Site**				0.229
CNS	1 (2%)	5 (13%)	6 (7%)	
Pleura/lung	15 (36%)	13 (33%)	28 (34%)	
Abdomen/ Retroperitoneal/ Pelvis	10 (24%)	13 (33%)	23 (28%)	
Extremity	8 (19%)	5 (13%)	13 (16%)	
Head/neck (not CNS)	7 (17%)	2 (5%)	9 (11%)	
Posterior trunk	1 (2%)	2 (5%)	3 (4%)	
**Presentation at Diagnoses**				0.108
Metastatic	1 (3%)	6 (15%)	7 (9%)	
Localized	38 (97%)	34 (85%)	72 (91%)	
**Surgery**				0.354
No	1 (2%)	3 (8%)	4 (5%)	
Yes	41 (98%)	37 (93%)	78 (95%)	
**Resection Status**				0.325
R0	31 (82%)	19 (68%)	50 (76%)	
R1	5 (13%)	8 (29%)	13 (20%)	
R2	2 (5%)	1 (4%)	3 (5%)	
**Chemotherapy**				0.009
Yes	5 (12%)	16 (40%)	21 (26%)	
No	37 (88%)	23 (61%)	60 (75%)	
**Radiation Therapy**				0.020
Yes	9 (21%)	19 (48%)	28 (34%)	
No	33 (79%)	21 (53%)	54 (66%)	
**History of Other Cancers**				1.000
Yes	11 (27%)	11 (29%)	22 (27%)	
No	30 (73%)	27 (71%)	57 (72%)	

CNS, central nervous system; HPF, high-power field. P < 0.05 indicates dependence between the characteristic and histological status.

### Treatment

All but one patient with benign disease had surgical intervention for their tumor due to a pre-existing diagnosis of metastatic prostate cancer, whereas 3 (8%) patients with malignant disease did not have surgery. Far fewer patients with benign histological status received chemotherapy and radiation therapy versus patients with malignant histology (see **Table 1**). Patients who received chemotherapy and those that did not undergo surgery had significantly worse outcomes. Patients with chemotherapy had a higher hazard rate than those without chemotherapy (P = 0.003, HR = 4.55, with 95% CI: 1.68–12.34), and patients without surgery had higher hazard rate than those who had surgery (P = 0.005, HR = 25.49, with 95% CI: 2.62–247.57). The types of chemotherapy and details of treatment given are outlined in **Table 2**. One patient who received sunitinib for treatment of renal cell carcinoma was not included in this table or analysis.

**Table 2:** An outline of the chemotherapy types and how many patients received each.

Type of Chemotherapy	Number of Patients who received these treatments as 1^st^ line	Number of Patients who received these treatments 2^nd^ line or later
Adriamycin/Ifosfamide	10	2
Taxane based*	3	4
Tyrosine Kinase Inhibitors (TKIs)**	1	2
Experimental^†^	1	1
Other^‡^	5	5
Patients who received multiple regimens	9	

*Taxane based regimens used: Carboplatinum/Taxotere, gemcitabine/docetaxol or taxotere, docetaxol, paclitaxel

**TKIs used: Imatinib, Sunitinib

^†^Experimental treatments used: GARFT inhibitor, anti-CD40 monoclonal antibody

^‡^Other treatments used: Dacarbazine, pemetrexed, Rapamycin, doxorubicin/cisplatinum, adriamycin/carboplatinum, cytarabine, avastin, cisplatinum, doxorubicin/ifosfamide

Note: Four of the five patients with benign histology who received chemotherapy had large tumors and two had incomplete resections

### Survival

**Fig 2:**
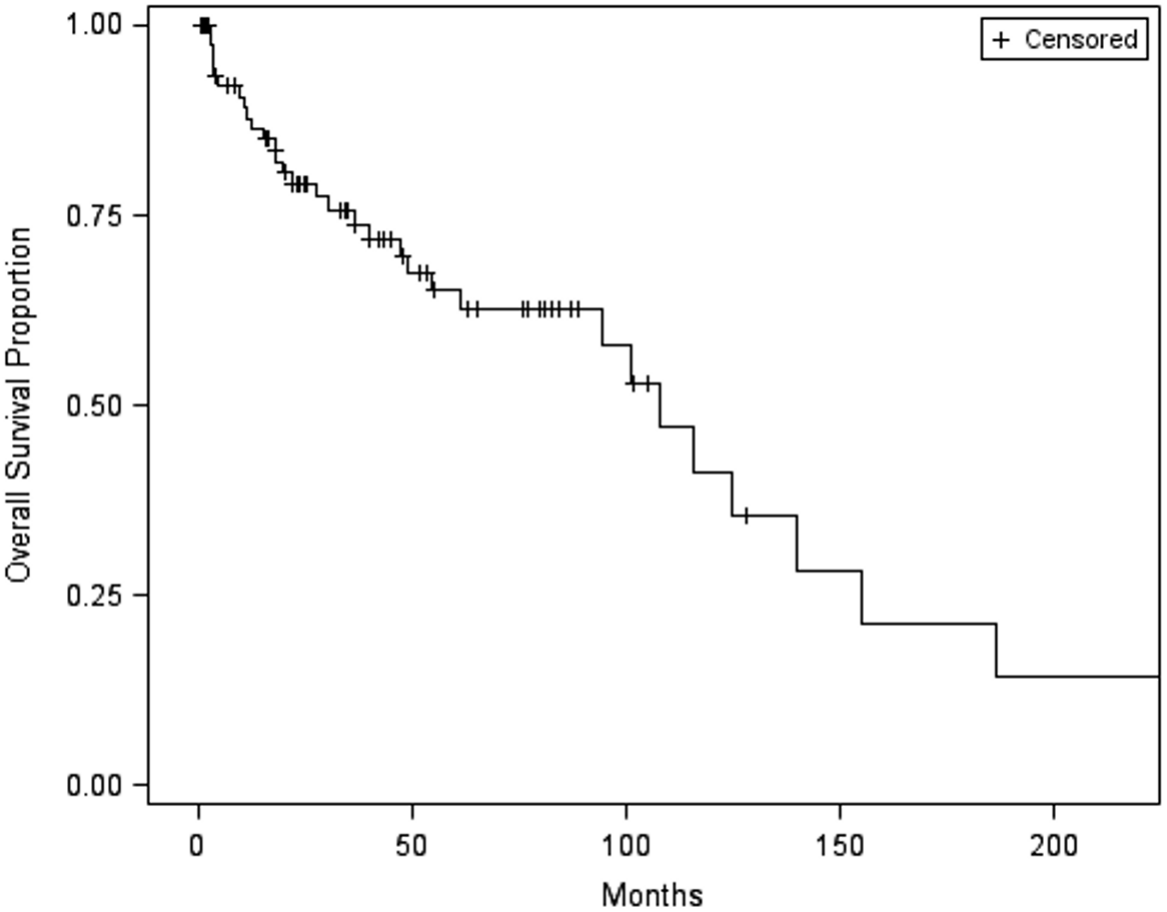
Overall survival curve of entire cohort.

**Fig 3:**
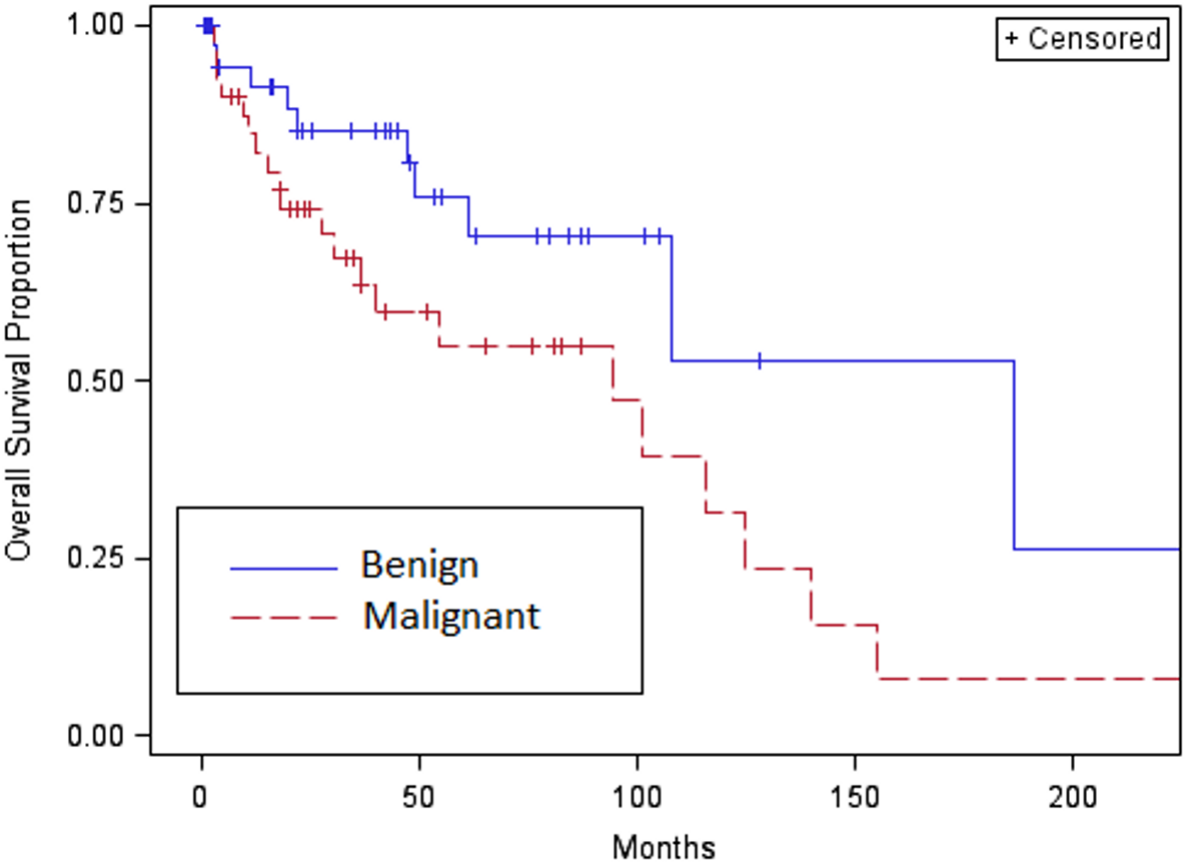
Overall survival curve of benign versus malignant.

Survival results are shown in **Table 3**. In our patient group (median follow-up of 55.3 months), the median overall survival for the entire cohort was 107.7 months (95% CI: 61.0 months to No Range) (survival curves shown in **Figs 2 and 3**). Thirty-two (39%) patients died of all-cause mortality. Although the overall survival for all patients at 1 year was 88%, only 65% of the patients were alive at 5 years. For patients with localized disease at diagnosis, the survival rates were shown to be similar. Patients with metastatic disease at diagnosis portend a worse prognosis, with a median overall survival of 54.6 months (95% CI: 3.4, 139.8). The 1-year survival rate for patients with metastatic disease at diagnosis was 69%, 51% at 3 years, and 26% at 5 years. Patients with benign SFT had a median overall survival of 186.6 months (61 months to NR). The 1-year survival rate was 91%, 85% at 3 years, and 76% at 5 years. Patients with malignant SFT had a median survival of 94.4 months (range, 30.6 to 124.9 months). The 1-year survival rate was 84%, 67% at 3 years, and 55% at 5 years.

**Table 3:** Point and interval estimation of the median overall survival and overall survival rates at 1, 3, and 5 years.

Survival Rate					Estimated Hazard Ratio (95%)
	Median Months	1 Year	3 Year	5 Year	
**All Patients**	107.7 (61.0, NR*)	87.8% (80.3%, 95.3%)	75.7% (65.6%, 85.9%)	65.1% (52.8%, 77.4%)	
**Histological Status**					
Benign	186.6 (61.0, NR)	91.3% (82%, 100%)	85% (72.9%, 97.1%)	75.8% (59.6%, 92%)	1
Malignant	94.4 (30.6, 124.9)	84.7% (73.4%, 96%)	67.3% (51.9%, 82.7%)	55% (37.1%, 72.9%)	22.2 (1.05, 4.70)
**Metastasis**					
Localized Diseases	107.7 (94.4, 154.9)	89.1% (81.5%, 96.7%)	77% (66.4%, 87.7%)	68% (55.5%, 80.6%)	1
Metastatic Diseases	54.6 (3.4, 139.8)	68.6% (32.1%, 100%)	51.4% (11.5%, 91.4%)	25.7% (0%, 66.6%)	2.24 (0.85, 5.92)
**Surgery**					
No	7.53 (3.43, 19.83)	25% (0%, 67.4%)	0 (NA)	0 (NA)	12.48 (3.79, 41.12)
Yes	107.7 (94.4, 154.9)	92.9% (86.9%, 98.9%)	80.2% (70.4%, 89.9%)	69% (56.5%, 81.4%)	1

*The upper bound is not estimable using the Kaplan-Meier product limit approach because of the large proportion of censoring (76.2%). Each hazard ratio is estimated based on univariable Cox regression.

NR = No Range

Patients with unresectable disease at diagnosis had a median overall survival of 7.53 months (95% CI: 3.4, 19.8). At 1 year, only 25% of patients with unresectable disease were alive, and none were alive at 3 years from diagnosis. Patients with resectable disease at diagnosis had a median overall survival of 107.7 months (95% CI: 61.0, 154.9). The 1-year survival rate for patients with resectable disease was 93%, 80% at 3 years, and 69% at 5 years.

By univariate analysis, only histologic status, complete resection, and use of chemotherapy impacted overall survival (P = 0.03, P = 0.01, and P<0.0001, respectively). Other factors, including primary site, metastasis at diagnosis, radiation use, concurrent second malignancy, tumor size, CD34 status and mitotic count, were not been found to be significantly associated with overall survival. In the multivariable analysis, surgery and chemotherapy remained significant, whereas others were not.

## Discussion

Although recent studies have provided much-needed molecular biological insight [17–19], there is a definite need to further understand both the clinical and molecular behavior of SFT. Central to this theme is the understanding of whether the histologic status (benign vs. malignant) affects outcome. This, in turn, can assist with selecting the appropriate population for which to conduct therapeutic studies. Thus, our group decided to review our institutional experience with benign and malignant SFT.

### Benign Is Not Always Benign

Patients with benign disease by pathology had a lower than expected survival rate. Four of these patients had concurrent, advanced secondary malignancies, discovered near the time of diagnosis of their SFT. One patient did not undergo surgery due to metastatic prostate cancer, and 7 had incomplete resections, 2 of which were R2 status due to tumor size. Additionally, 11 patients had recurrent disease, 5 of these being distant. Histology was not independent of certain characteristics such as chemotherapy, as seen in Table 1 (P = 0.009), indicating that it can be confounded with other covariates. This highlights the limitations of microscopic diagnosis, with the hope that future molecular markers will help to better clarify benign versus malignant disease.

### Histological Status Remains Important

With regard to survival, it seems clear that patients with localized disease or surgically resectable disease have a superior survival compared to patients with metastasis at diagnosis or unresectable disease. Our study also confirms the importance of the histologic designation and survival. Patients with benign SFT appear to have better outcomes than patients with malignant SFT.

### Secondary Malignancies

An unexpected observation is the high percentage (27%) of patients harboring other malignancies at some point during their lifetime. There was no statistical difference between histologic subtypes. Of the 22 patients afflicted with a second malignancy, 5 patients had ductal carcinoma of the breast and 5 had adenocarcinoma of the prostate. This association could have been random. However, two patients also had pancreatic cancer and one had renal cell carcinoma. One of the patients with pancreatic cancer led to the decision to not resect the SFT.

### Our Center’s Experience with Treatment of SFT

Although the vast majority of our patients underwent surgery, those who did not had very poor outcomes, with no patients surviving to 3 years after diagnosis. Radiation therapy did not affect overall survival. Of interest, patients treated with chemotherapy had an inferior overall survival compared to untreated patients; this likely represents treatment bias, since chemotherapy preselected patients who were clinically aggressive. For example, four of the five patients with benign histology who received chemotherapy had large tumors, two of which had incomplete resections. In a study of 21 patients, conventional chemotherapy was shown to often have a temporary stabilizing effect on the growth of advanced or metastatic SFT [21]. Due to the retrospective nature of our study, it is not feasible to accurately assess response to chemotherapy. Regardless, It should be recognized that despite the myriad of therapies attempted, no objective responses were documented in any patients. However, some patients received the same therapy beyond first assessment, which suggests that disease stability may have been present prior to eventual progression.

The survival rate for our entire cohort of 82 patients was 65% at 5 years, with a median OS of 107.7 months, which is lower than what has been typically seen in this malignancy. For the 42 patients with benign tumors, survival was 76% at 5 years, while the 40 patients with malignant tumors had a survival of 55% at 5 years. In total, 16 patients had incomplete resections, 22 patients had other cancers (4 of which were advanced stage in patients with benign SFTs), and 7 patients presented with metastatic disease upon diagnosis. In comparison to other studies, Spitz FR et al had 36 patients, 5 year OS was 71% and only 13 had recurrences. Magdeleinat P et al had 60 patients, however, all had pleural SFTs with an OS of 97% in benign and 89% in malignant. van Houdt WJ et al had 81 patients with a 5 year OS of 84% after excluding patients that had unresectable tumors, only 19% of these patients had a high mitotic rate. [9, 20, 22] The majority of other studies surveyed involved mostly benign, pleural based SFTs with complete surgical resection, with survival rates ranging from 54% to 89% at 10 years. Our review contained more patients with malignant tumors in diverse anatomic locations, many of which had incomplete resections, as well as patients with benign tumors by pathology that displayed malignant behavior and patients with benign histology that unfortunately succumbed to comorbidities such as other malignancies.

### Understanding the Biology of SFT

As seen with previous studies, malignant SFT was associated with larger, mitotically active tumors that were more likely to be metastatic at diagnosis compared to benign SFT. We also determined that the primary site did not favor benign histology over malignant histology. This could be explained by the sample size of each primary site. Whether this implies that the malignant variant represents an evolution of a benign SFT that undergoes molecular alterations resulting in a more aggressive phenotype is uncertain. Recent studies have demonstrated recurrent NAB2-STAT6 gene fusions in both benign and malignant SFT by integrative sequencing. [18, 19] Both Mohajeri et al. and Robinson et al. identified this gene fusion in the vast majority of the SFTs that they studied. [8, 18, 23] Further studies are warranted to identify the additional biomarkers, as well as potential pharmaceutical targets for the malignant subtype. Unfortunately, due to the retrospective nature of our study, we were unable to perform NAB2-STAT6 analysis on our samples.

## Conclusions

As with any retrospective study, there are limitations. First, our tertiary care facility is a referral center for sarcoma patients; thus, the lower than-expected outcomes could reflect the advanced nature of the disease presentations. This explains why our study had higher numbers of malignant cases (49%) than reported in the literature (12–22%). Second, our pathology review was not blinded, so it is possible to introduce bias. Additionally, a microscopic pathological evaluation has its limitations in predicting the clinical behavior of this disease, so molecular markers could further help to predict survival as well as propensity for metastasis. These markers could also potentially provide targets for drug development.

In conclusion, we present a large series of SFT patients treated at one center with an attempt to correlate pathologic features with clinical outcomes. Our analysis demonstrates that malignant SFT portends a worse prognosis than the benign counterpart. Importantly, surgical extirpation has a clear role regardless of histologic subtype. Because current systemic therapy regimens do not seem to be effective, it is clear that better systemic therapies are needed for patients with metastatic/unresectable disease.

## Supporting Information

S1 Dataset De-identified patient data. Contains patient demographics, survival and appropriate descriptors used for statistical analysis. Dates were removed to preserve patient anonymity.
(XLS)
